# Anticipatory prescribing in community end-of-life care: systematic review and narrative synthesis of the evidence since 2017

**DOI:** 10.1136/spcare-2022-004080

**Published:** 2023-05-26

**Authors:** Ben Bowers, Bárbara Costa Pereira Antunes, Simon Etkind, Sarah A Hopkins, Isaac Winterburn, Isla Kuhn, Kristian Pollock, Stephen Barclay

**Affiliations:** 1 Department of Public Health and Primary Care, University of Cambridge, Cambridge, Cambridgeshire, UK; 2 Queen's Nursing Institute, London, UK; 3 Department of Psychiatry, Cambridge University, Cambridge, Cambridgeshire, UK; 4 School of Clinical Medicine, Cambridge University, Cambridge, Cambridgeshire, UK; 5 School of Health Sciences, University of Nottingham, Nottingham, UK

**Keywords:** clinical decisions, drug administration, home care, pain, symptoms and symptom management, terminal care

## Abstract

**Background:**

The anticipatory prescribing of injectable medications is recommended practice in controlling distressing symptoms in the last days of life. A 2017 systematic review found practice and guidance was based on inadequate evidence. Since then, there has been considerable additional research, warranting a new review.

**Aim:**

To review the evidence published since 2017 concerning anticipatory prescribing of injectable medications for adults at the end-of-life in the community, to inform practice and guidance.

**Design:**

Systematic review and narrative synthesis.

**Methods:**

Nine literature databases were searched from May 2017 to March 2022, alongside reference, citation and journal hand-searches. Gough’s Weight of Evidence framework was used to appraise included studies.

**Results:**

Twenty-eight papers were included in the synthesis. Evidence published since 2017 shows that standardised prescribing of four medications for anticipated symptoms is commonplace in the UK; evidence of practices in other countries is limited. There is limited data on how often medications are administered in the community. Prescriptions are ‘accepted’ by family caregivers despite inadequate explanations and they generally appreciate having access to medications. Robust evidence of the clinical and cost-effectiveness of anticipatory prescribing remains absent.

**Conclusion:**

The evidence underpinning anticipatory prescribing practice and policy remains based primarily on healthcare professionals’ perceptions that the intervention is reassuring, provides effective, timely symptom relief in the community and prevents crisis hospital admissions. There is still inadequate evidence regarding optimal medications and dose ranges, and the effectiveness of these prescriptions. Patient and family caregiver experiences of anticipatory prescriptions warrant urgent investigation.

**PROSPERO registration:**

CRD42016052108

**What is already known about this topic**

- Anticipatory prescribing has become established good practice in controlling distressing symptoms for patients dying in the community.
- Current anticipatory prescribing guidance and practice is based on an inadequate evidence base.

**What this study adds**

- The prescribing of anticipatory medications is a significant event for patients and families and signifies the imminence of death.
- There remains inadequate evidence to draw conclusions about the impact of anticipatory prescriptions on symptom control or crisis hospital admissions.

**How this study might affect research, practice and policy**

- Robust research is needed to investigate the clinical effectiveness, cost-effectiveness, safety and acceptability of anticipatory prescribing.
- Patient and family caregiver experiences of anticipatory prescriptions and their involvement in decisions to administer medication require urgent investigation.

## Introduction

Anticipatory prescribing of injectable medication is considered essential for timely management of distressing last-days-of-life symptoms in the community.^1–5^ A key feature of anticipatory prescribing is that the medications are prescribed ahead of possible need: ‘just in case’. Medications are typically prescribed for symptoms of pain, shortness of breath, agitation, nausea and vomiting and noisy respiratory tract secretions.^2 6 7^ Anticipatory prescriptions include controlled drugs and those with the potential for misuse including opioids and benzodiazepines. These are dispensed and an accompanying prescription and administration authorisation chart is completed. Once this is done, permission has been granted for nurses, paramedics and general practitioners (GPs) to use (administer) the medications subcutaneously based on their clinical assessment that the person is dying and needs them for symptom management. The chart details the medications, indications and dose, typically with dose ranges that enable some discretion when using the drugs. In some countries, including Australia and parts of the UK, appropriately trained family caregivers (family and friends) can administer these injectable medications with support. Administration may be with a needle and syringe or needle-free technique, where the syringe is connected to a pre-existing subcutaneous catheter.^8 9^

Anticipatory prescribing is actively promoted in end-of-life care guidance documents internationally,^1 2 4 10–12^ in part recently due to the rapid increase in the numbers of terminally ill patients dying at home and in care homes during the COVID-19 pandemic.^13 14^ The intervention has become an embedded part of symptom control care and is widely perceived by clinicians and policymakers to be a key end-of-life clinical intervention.^15–20^ However, two UK independent reviews into the mismanagement of injectable medications have raised serious concerns about inappropriate practices in prescribing and using anticipatory medications.^21 22^

Four years ago, we systematically reviewed and synthesised evidence supporting the practice of anticipatory prescribing for dying adults in the community, and found that it has been founded on an inadequate evidence base.^23^ Care was based primarily on the belief of doctors and nurses that access to these medications reassures patients and their families, effectively controls symptoms and prevents crisis hospital admissions.^7 24–26^ There was no reliable data on how often medications are prescribed or subsequently used in the community. There was inadequate evidence to allow conclusions to be drawn about anticipatory prescribing in terms of its cost-effectiveness, safety, impact on patient-reported symptoms or prevention of crisis hospital admissions.^23 27 28^ No studies had examined patient views and experiences of anticipatory prescribing. Studies of family caregiver opinions were limited to evaluations of family carer administration of injectable medications.^28 29^ In summary, there was paucity of high-quality research to inform care.

Since our original review (search undertaken in 2017), a considerable amount of new research has been conducted to develop the anticipatory prescribing evidence base, not least due to the challenges of the COVID-19 pandemic. A synthesis of this new body of research is warranted to determine how knowledge has advanced.

## Aim

We aimed to synthesise the evidence published concerning anticipatory prescribing of injectable medications for adults at the end-of-life in the community, to inform practice and guidance. We included papers published from May 2017 onwards, building on our original systematic review.^23^

The focus of our review is exclusively on injectable medications, as this is the most widespread form of anticipatory prescribing, requires specific training and has been highlighted to have potential for misuse.^1 21 30^

## Review questions

Regarding the anticipatory prescribing of injectable medications for adults in the community approaching the end of their lives:

1. What is current practice?
2. What are the attitudes of patients?
3. What are the attitudes of family caregivers?
4. What are the attitudes of community healthcare professionals?
5. What is its impact on patient comfort and symptom control?
6. Is it cost-effective?

## Methods

We conducted a systematic review and narrative synthesis^31 32^ of empirical evidence published since May 2017. The review protocol was registered with PROSPERO (reg. no. CRD42016052108). We use the same research questions and methods (including search strategy, eligibility and data synthesis approaches) as our original review to allow ease of comparison for clinicians, researchers and policymakers. We report the new evidence published since May 2017 in the results and narrative synthesis. In the discussion section, we draw comparisons to what our original review found to highlight how the evidence base has evolved, and to identify what the new empirical evidence adds to the existing knowledge base.

The search strategy was developed and refined with the review team’s Information Scientist (IK). The search was conducted using nine databases: Medline, CINAHL, Embase, Web of Science, PsycINFO, Cochrane Library, Social Care Online, HMIC and King’s Fund. Box 1 details the Boolean search strategy used in Medline; searches in the other databases were adapted from this strategy (online supplemental material 1).

10.1136/spcare-2022-004080.supp1 Supplementary data

All databases were searched from 1 May 2017 to 1 March 2022. The digital archives for BMJ Supportive & Palliative Care and Palliative Medicine were also hand-searched for published papers from May 2017 to March 2022. These two journals were chosen as they contained for the largest number of eligible studies from the initial search strategy and from our original review. Reference and citation searches of all included papers were undertaken.

Box 1 **Medline search strategy**

Epub Ahead of Print, In-Process & Other Non-Indexed Citations, Ovid MEDLINE(R) Daily and Ovid MEDLINE(R) first May 2017 to first March 2022

((palliative adj medicine adj kit*) or (liverpool adj care adj pathway*) or ((end adj2 life) adj2 ((care adj plan*) or (care adj pathway*))) or (gold adj standard* adj framework*) or ((prescrib* or prescription* or medicat* or medicine* or drug* or pharma or pharmaceutical* or packet* or pack* or pak* or box* or kit* or (care adj plan*) or (core adj “4”) or (core adj four)) adj3 (crisis* or comfort* or anticipate* or anticipatory or anticipation or preemptive or pre-emptive or (just adj in adj case) or PRN or (pro adj re adj nata) or (as adj required)))).ti,ab.

AND

(exp Terminal Care/ or exp Palliative Care/ or exp “Hospice and Palliative Care Nursing”/ or exp death/ or exp Palliative Medicine/ or exp Terminally Ill/ or ((end adj2 life) or ((final* or last*) adj1 (hour* or day* or minute* or week* or month* or moment*)) or palliat* or terminal* or (end adj stage) or dying or (body adj2 (shutdown or shut* down or deteriorat*)) or deathbed).ti,ab.)

The Boolean search strategy for all databases is available in online supplemental material 1.

Papers were included if they presented new empirical data on the anticipatory prescribing of injectable medications for end-of-life symptom control in the community, published in English. The review was limited to papers where patient participants were aged 18 years and over. Published case studies, audits and conference abstracts were included, mirroring the inclusion criteria for our original systematic review.^23^ Studies were excluded if they reported on the reactive prescribing of injectable medication after symptoms occurred. Box 2 details the review inclusion and exclusion criteria.

Box 2 **Review inclusion and exclusion criteria**

**Inclusion criteria:**

- Published papers presenting empirical research on the prescribing of injectable medications ahead of need to control terminal symptoms for adults (aged 18 years and over).
- Participants receiving care at home in the community (including nursing and residential home care settings).
- Peer-reviewed quantitative and qualitative studies, case studies, audits, published conference abstracts.
- Key areas for data extraction:
  - Descriptions of current practice.
  - Patient-reported views and experiences.
  - Family caregivers reported views and experiences.
  - Community-based healthcare professional reported views and experiences.
  - Patient comfort/symptom control (reported by whom).
  - Evidence for cost-effectiveness, including impact on:
    - Admission avoidance.
    - Place of death.
    - Healthcare activity,
    - Cost of medications.
- Studies published between 1 May 2017 and 1 March 2022.
- English language full text.

**Exclusion criteria:**

- Anticipatory prescribing in non-terminal care situations.
- Prescriptions that do not include injectable medication.
- Studies concerning the reactive prescribing of injectable medication after symptoms occurred, including via syringe pump/driver (continuous subcutaneous infusion).
- Children (aged 17 years or under).
- Prescribing in hospital, hospice or prisons.
- Papers presenting no new empirical data for example, editorials, opinion papers or narrative reviews.
- Research examining assisted dying or euthanasia.
- Research examining continuous sedation until death.
- Grey literature.

Taken from ‘Anticipatory prescribing of injectable medications for adults at the end of life in the community: A systematic literature review and narrative synthesis.’^23^ SAGE Publishing.

Search results were uploaded into Endnote X9 and duplicates removed. BB screened titles for eligibility using the inclusion/exclusion criteria. After exclusion of irrelevant and duplicate titles, abstracts were independently screened for eligibility by three reviewers (BB, BCPA, SAH), with disagreements resolved by consensus. Full-text papers were assessed for eligibility by BB, with a second review by BCPA or SAH, where eligibility was uncertain. From the 2379 records identified, 10 papers met the inclusion criteria. Reference, citation and hand searches identified a further 18 papers; of which, 13 were published conference abstracts not registered in databases.

A review-specific data extraction form was used to record participant characteristics and methods from each included paper, and the results relevant to each of the six review questions (online supplemental material 2). In tandem to the data extraction process, two members of the review team (BB, BCPA, SAH, SE and IW) independently assessed each paper in terms of its internal validity, appropriateness and contribution in answering the review questions, using a review-specific version of Gough’s Weight of Evidence (WoE) criteria (box 3).^33^ This modified version had been successfully used in our original systematic review. Where one or more of the reviewers were an author of an included study, two non-authors conducted the WoE assessment. Discrepancies in assessment decisions were discussed between reviewers and final scores were agreed through consensus.

10.1136/spcare-2022-004080.supp2 Supplementary data

Box 3 **Review-specific Gough’s ‘weight of evidence’ criteria**

**WoE A** is the assessment of internal validity: whether the study design was rigorous; whether this could be adequately assessed from a transparent, comprehensive and repeatable method; accurate and understandable presentation and analysis; if samples and data collection tools were appropriate to the aims of the study and whether conclusions flowed from the findings and are proportionate to the method. Papers are scored as high/medium/low.

**WoE B** relates to the appropriateness of the study design to the six review specific questions. Papers are scored as high / medium / low.

- Review questions 2, 3 and 4: inductive research designs interpreting the views directly reported by patients/family caregivers/healthcare professionals=high.Deductive research designs interpreting the views directly reported by patients/family caregivers/healthcare professionals=medium.Deductive research designs indirectly interpreting the views of patients/family caregivers/healthcare professionals=low.
- Review questions 1, 5 and 6: the fitness for purpose of that form of evidence for answering the questions were made on a paper-by-paper basis.

**WoE C** relates to detailed judgements about each study relating to the relevance of the focus of the evidence for answering the review questions. This includes any sampling issues relating to the interpretation of the data; whether the study was undertaken in an appropriate context from which results can be generalised to answer the relevant review specific questions. Papers are scored as high/medium/low.

**WoE D** is the extent that a study contributes evidence to answering the review questions. The above three sets of judgement scores are combined to give the overall ‘Weight of Evidence’ as high/medium/low.

Reprinted from ‘Anticipatory prescribing of injectable medications for adults at the end of life in the community: A systematic literature review and narrative synthesis.’^23^ SAGE Publishing. Review specific criteria were adapted from^33^ reprinted by permission of the author and publisher Taylor & Francis Ltd.

Extracted data were entered into Excel to aid the narrative synthesis of the included papers.^31 32^ An inductive narrative synthesis approach was chosen for its applicability in interpreting and integrating heterogeneous study methods and qualitative and quantitative evidence.^32^ The following three iterative stages were involved, replicating the approach adopted in our original review:

1. Study descriptions and results were tabulated based on the sample population, methods and the research questions the results answered.
2. BB, a clinical academic palliative care nurse with experience in conducting narrative syntheses, completed an inductive narrative analysis to identify the main, recurrent and divergent evidence across the studies in answering each research question.^31 32^ The similarities and differences between the studies, including methodological approaches and research methods, context, characteristics of participants and findings were considered throughout the iterative inductive synthesis. The significance and applicability of findings from studies conducted by researchers from different disciplinary and epistemological positions were debated with BCPA, KP and SB; consensus in the synthesis was reached. The synthesis was further refined through discussion of the review results and their implications with the Cambridge Positive Ageing and Cambridge Palliative and End-of-Life Care Patient and Public Involvement Groups, the review team, clinicians and interested members of the public (including people with experience of being family caregivers) through a series of presentations.
3. The WoE score of papers informed the synthesis. Papers judged as having an overall high WoE (D) score were considered more robust and appropriate in answering the review questions than papers with a medium score in the synthesis.^32 33^ Results from papers with an overall low WoE score were regarded as inadequate to draw conclusions from unless they supported the findings of papers rated as WoE medium or high.^32^ We included papers with an overall low WoE score in the synthesis to demonstrate the overall evidence base, highlighting the gaps in knowledge and the need for future research.

## Results

**Figure 1:**
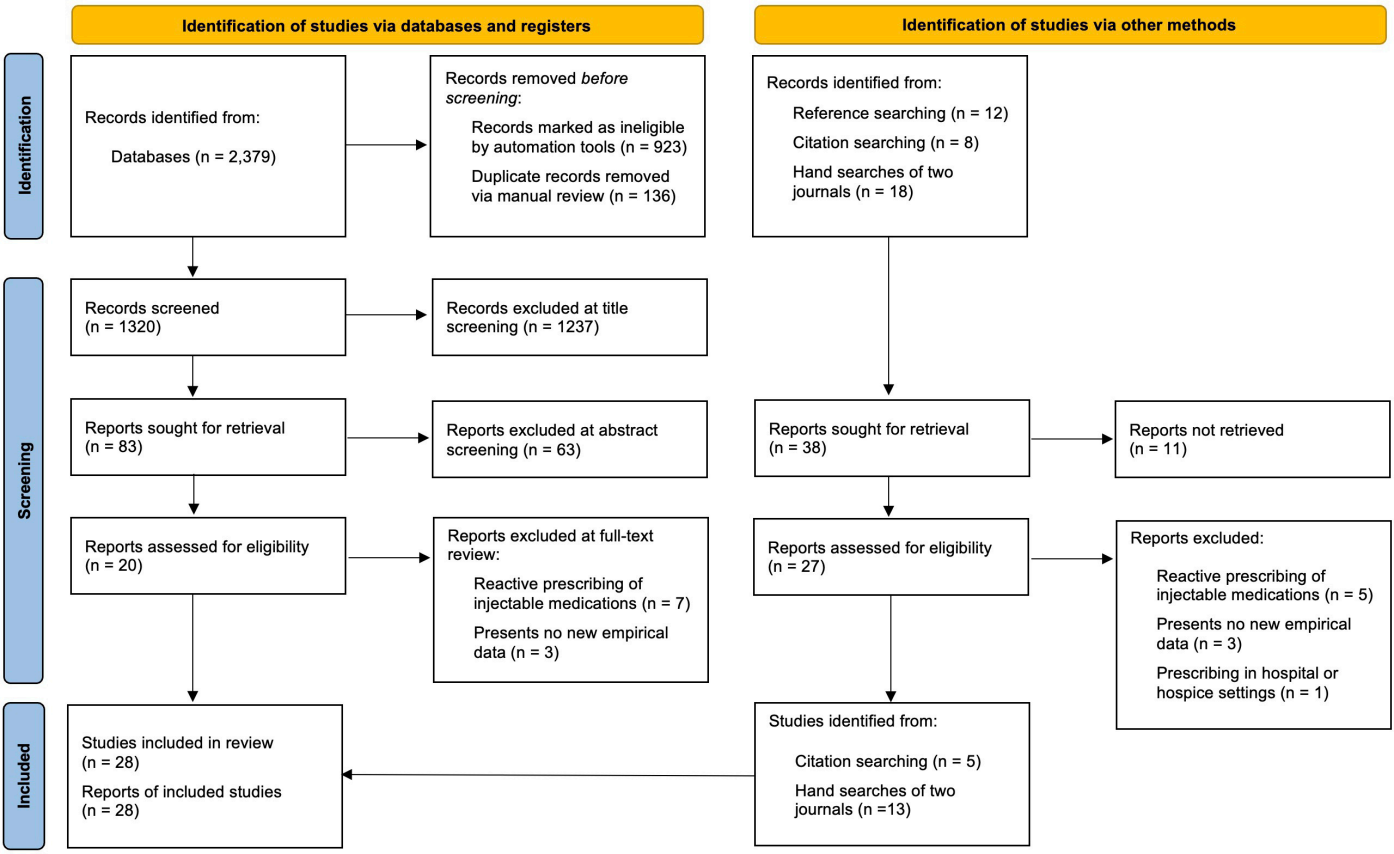
Preferred Reporting Items for Systematic Reviews and Meta-Analyses diagram. Adapted from ‘The PRISMA 2020 statement: an updated guideline for reporting systematic reviews’ (Page *et al* 2021).^34^ BMJ Publishing Group Ltd.

Figure 1 details the Preferred Reporting Items for Systematic Reviews and Meta-Analyses flow diagram^34^ of the review and the number of papers that met the criteria for inclusion.

A total of 28 papers, reporting on 27 studies, were included in the synthesis: 14 research papers and 14 conference abstracts. Different elements of one study were reported in two papers^6 35^: both papers were treated as individual study units in the analysis as they presented different findings. Papers reported on practice in the UK (n=19), Australia (n=5), Australia and New Zealand (n=1), Canada (n=1), British Isles (n=1) and Norway (n=1). Published papers’ methods included qualitative interviews with healthcare professionals (n=9), qualitative interviews with family caregivers (n=5), retrospective patient notes reviews (n=10), staff or family questionnaires (n=5), clinical audits (n=2), guidance document analysis (n=2), qualitative interviews with patients (n=1), a randomised pilot trial (n=1) and quasirandomised control trial (n=1); eight papers reported on multiple-methods, including those mentioned above. Online supplemental material 3 summarises the included papers and their overall WoE: 6 were rated as high, 17 medium and 5 low.

10.1136/spcare-2022-004080.supp3 Supplementary data

### What is current practice?

Twenty-one studies have reported on anticipatory prescribing practice in the community: these were rated as WoE low to high and conducted primarily in the UK and Australia.^5 6 8 9 17 19 36–50^ Surveys of healthcare professionals and analysis of governance documents, predominately rated as WoE low and medium, suggest that anticipatory prescribing is widespread established end-of-life practice in the UK,^19 36^ with some services also permitting the prescribing of anticipatory syringe pumps/drivers (continuous subcutaneous infusions).^5 19 37 38^ Anticipatory prescriptions appear to be less commonplace in Australia, Canada and Norway, with the intervention focused primarily on supporting populations with a terminal cancer diagnosis.^17 17 39–41^

#### Prescribing practices

The frequency of anticipatory prescribing in the community has been investigated by five studies^6 17 42–44^ rated as WoE low to high. Prescribing rates vary from 14% to 96% of deaths in the community (home or care home), dependant on underlying terminal conditions, geographical location and community healthcare services involved.^6 17 42–44^ However, data are often limited by focusing on populations receiving specialist palliative care or inadequate definitions of anticipatory prescribing.^17 42 44^ Two studies, assessed as WoE medium and high, utilised deceased patient records in general practice populations and identified a prescribing rate of 44%^43^ and 51%.^6^ The likelihood of being issued an anticipatory prescription is significantly higher for patients with a recorded preferred place of death (OR: 34; 95% CI: 15 to 77; p<0.001) and for patients receiving specialist palliative care involvement (OR 7; 95% CI: 3 to 19; p<0.001).^6^

Anticipatory prescriptions are initiated independently by GPs or at the request of community nurses and specialist palliative care teams in the UK and Norway.^6 39 45^ In parts of Australia, prescriptions are reliant on nurses prompting GPs to consider doing so^17 40 46^; two Australian studies, both rated as WoE low, identified that a referral to specialist palliative care services triggered a standard request for GPs to initiate prescribing.^17 46^

There is considerable variation in the timing of anticipatory prescribing prior to death.^6 43^ Prescriptions range from 0 to 1212 days before death,^6^ with a median timing of 14 to 22 days before death for patients with terminal cancer conditions and 6–12 days for those with non-cancer conditions.^6 43^ Three UK studies, rated as WoE high, found prescribing clinicians issued prescriptions well in advance of anticipated need, even if they were unlikely to be used^6 45 47^; the presence of prescriptions in the home are used as a sign to alert other visiting clinicians to the terminal nature of the patient’s condition.^45^

The standardised prescribing of four injectable medications for symptoms of pain, nausea and vomiting, agitation and noisy respiratory tract secretions is commonplace in the UK and prompted by local and national guidance.^5 6 42 44 45 48^ Two UK studies, rated as WoE medium and high, found anticipatory medications to start via syringe pumps/drivers were prescribed for between 29% and 33% of patients alongside individual ‘as required’ injections in the community.^6 37^ This practice is dependent on clinician preferences and local healthcare cultures,^5 6 38 45^ and has not been reported on in other countries. Opioids are commonly prescribed for pain control in the UK and Australia^5 40 44 48 49^; the sedative midazolam is frequently prescribed for agitation.^5 6 40 42 48^ Specialist palliative care clinicians report prescribing midazolam and opioids in higher doses when anticipating a possible catastrophic terminal event, such as bleeding or airway obstruction.^49^

#### Administration practices

The literature concerning the use of prescribed anticipatory medications remains limited. GPs typically rely on visiting nurses and paramedics to assess when to start medications and update them about their use.^39 40 44 45^ Four studies, rated as WoE medium to high, reported that between 37% and 64% of anticipatory prescriptions were used^44 47 48 50^; these studies refer to small numbers of patients, often receiving specialist palliative care. Six studies identified that medications are most frequently used for symptoms of pain, agitation and nausea and vomiting.^5 9 40 43 48 50^ These studies relied on recollections of medications used or incomplete records, limiting the reliability of data.

There is insufficient published research regarding the timing of first medication use prior to death. One small-scale study,^43^ rated as WoE medium, found patients with a diagnosis of cancer, frailty or dementia first received medications a median of 4 days before death. A service evaluation,^44^ rated as WoE medium, reported the median timing between prescription and first drug administration was 9 days for patients with cancer (range 0–368 days), and 61 days for those with non-cancer conditions (range 3–298 days). Both studies were limited by having partial access to patient records.

The time from nurses receiving a family request to administer medication to giving the dose can vary greatly,^9 39 50^ with a median time of 105 min reported in one UK multisite study rated as WoE high.^9^ Two Australian and one UK paper have reported on initiatives to train family caregivers to assess symptoms and give injectable medication, with or without direct clinical guidance.^8 9 46^ A survey of healthcare professionals working in the UK and Ireland, undertaken at the start of the COVID-19 pandemic and rated as WoE high, reported that numerous community healthcare services were considering this option, in anticipation of end-of-life care needs overwhelming community healthcare resources.^19^ The extent to which these policy changes have been put in place in practice, or will persist after the pandemic, remains unclear.

### What are the attitudes of patients?

Only one published study has directly investigated patients’ attitudes regarding anticipatory prescribing. The interview study,^47^ rated as WoE high, reported the views of six case study participants where anticipatory medications had been prescribed but not yet used: it was unclear how many of these participants were patients or family members. The prescribing of anticipatory medications was a significant event for patients and clearly signified the imminence of death.^47^ No published studies have investigated patients’ views and experiences of the administration of prescribed anticipatory medications.

### What are the attitudes of family caregivers?

Five studies have investigated family caregiver perspectives regarding nurses overseeing and administering prescribed medications: this is standard practice in the UK and several other countries.^47 50–53^ Two qualitative interview studies, rated WoE medium to high, identified anticipatory prescriptions were ‘accepted’ into the home by family caregivers despite receiving inadequate explanations about medications, often because symptoms of suffering were expected at the end-of-life.^47 51^ A survey of bereaved family caregivers (n=38) and two interview studies (n=18 and n=2), rated as WoE medium, found that anticipatory prescriptions were generally viewed as being helpful.^50 52 53^ However, an unknown number of family caregivers reported feeling distressed when they realised that the prescription indicated that death was imminent.^50 52^ Family caregivers expressed concerns about storing controlled drugs in the house,^47 52^ experienced difficulties in getting nurses to visit to administer the medications in a timely manner^50 52^ or expressed ambivalence regarding the helpfulness of medication.^51^ Four of the five studies were only reported briefly in conference abstracts.^50–53^ Overall, it is not clear from the available studies if anticipatory prescribing was reassuring, a cause for concern or both.

Family caregiver attitudes are clearer regarding initiatives where they administer (give) the injections.^8 9 46 54^ Four UK and Australian studies, rated as WoE low to high, found that family caregivers reported the training^46 54^ and experience of administering anticipatory medications to be acceptable.^8 9^ Self-reported confidence in administering injections increased with practice.^8 46^ However, a service improvement initiative, rated as WoE low, specified that relatively few family caregivers were willing or able to take on the role.^46^ A UK multicentre randomised pilot trial,^9^ rated as WoE high, found that family caregivers selected by patients to give their injectable medications tended to have a healthcare background; most family caregivers struggled to recognise the difference between symptoms and worried about accidentally hastening death by giving injections. Despite these concerns, family caregivers reported that being able to administer injections increased feelings of empowerment and control; the median time taken for family caregivers to administer injections was 5 min.^9^

### What are the attitudes of community healthcare professionals?

The range of views of healthcare professionals towards anticipatory prescribing are reported in 15 studies of community nurses, palliative care nurses, care home staff, pharmacists, GPs and palliative doctors in the UK, Australia, New Zealand and Norway (five rated as WoE high, eight medium and two low).^5 9 17 19 37 39 40 42 45–47 49 53 55 56^ The majority of the studies focused on the perceptions of GPs and nurses.^9 17 39 40 42 46 47 53 56^ No published studies have investigated emergency ambulance paramedics’ views and experiences.

Healthcare professionals’ views are largely positive towards anticipatory prescribing. GPs and nurses perceive the intervention provides proactive and effective symptom control, helps prevent crisis hospital admissions and reassures patients, families and clinical teams.^9 17 37 39 40 45 46 49 56^ One survey study, rated as WoE medium, found palliative care doctors are uncertain whether anticipatory prescriptions for catastrophic terminal events are beneficial, as patients often die before medication can be given or take effect and conversations about unlikely events may cause disproportionate and unnecessary anxiety.^49^ However, doctors prescribe medications out of concern that patients may experience considerable distress if they are not available.^49^ The lack of robust evidence and guidance to inform this practice is a concern for palliative care doctors.

Key components of successful anticipatory prescribing are identified in 11 studies with healthcare professionals, rated as WoE low to high. Effective communication and close partnership working between GPs and community nurses are perceived to be vital in effective prescribing and timely medication administration; mutual respect for each other’s skills, expertise and ease of access to each other are considered essential.^39 40 45 46 56^ Healthcare professionals appreciate standardised systems and local policies that prompt timely prescribing and recommend safe starting doses.^17 42 45 53 55^ Two studies, both rated WoE high, identified that GP and nurse-led anticipatory prescribing conversations with patient and families are initiated in a way intended to lessen worries about the medication and their potential symbolic significance, while ensuring prescriptions are accepted.^45 47^ The ready availability of stock in community pharmacies is considered vital in ensuring prescriptions are dispensed in a timely way.^19 40^

Some prescribers are wary about the safety of anticipatory prescribing, especially prescribing strong opioids ahead of need since they remain accountable for drug errors or misuse.^17 40 45^ GPs were reluctant to leave controlled drugs in the home if there was a history of drug misuse in the family^40 45^ and rely on visiting nurses to monitor potential risks.^39 45^ Concerns about medication wastage can also be a barrier to prescribing.^19^

The administration of anticipatory medications raises safety concerns for healthcare professionals. For example, deciding when to administer medication causes less experienced nurses considerable unease, and some nurses report that they lack the confidence to initiate injections or adjust doses.^39 40^ One interview study, rated as WoE high, identified that nurses value clear instructions on when to administer the medication and what doses to give.^39^ Doctors and nurses report adverse patient events have occurred when medications were administered without an adequate clinical assessment of need or the wrong medication was given.^37 40 49^ Views on the safety of prescribing anticipatory syringe pumps/drivers are divided: some consider them vital in ensuring timely symptom control,^5 37^ while others view it as unsafe practice that can lead to inappropriate medications and doses being initiated.^37^

Some nurses and GPs think it too burdensome on family caregivers to train them to administer injectable medication, especially if families express concerns about accelerating death by giving medication.^9^ Nurses are cautious and selective about who they approach to take on this role.^9 46^

### What is its impact on patient comfort and symptom control?

The impact of anticipatory prescribing on patient comfort and symptoms has been reported in two studies.^9 50^ A survey of bereaved family caregivers,^50^ rated as WoE medium, found that just over half of the respondents reported that the medication was used, usually for pain or agitation, with good effect. Among the patients who required medications, problems family caregivers reported included deciding when to call for help and delays in clinicians attending to administer medication. A randomised pilot trial,^9^ rated as WoE high, found family carer and healthcare professional-reported patient symptom scores improved after the administration of injectable medications; however, there was considerable missing data on reported comfort before and after injections when family caregivers administered these. No published studies have reported on patient perceived comfort and symptom control.

### Is it cost-effective?

Anticipatory prescribing is a relatively low-cost intervention. Two UK records review studies, both rated as WoE medium, have calculated the costs of prescriptions. The first study identified the median cost of prescriptions as £43.17 per patient^35^; the second study calculated a prescription cost of £50 per patient.^48^ Both studies identified that haloperidol, frequently prescribed for possible symptoms of nausea and vomiting, accounted for much of these costs. The median cost of administered medications was £2.16 per patient, resulting in substantial drug wastage costs.^35^

The relationship between anticipatory prescribing and subsequent service use remains unclear.^41^ One population-level and retrospective cohort study, rated as WoE medium, has measured the impact of anticipatory prescribing on service use.^41^ The study identified that anticipatory prescribing alongside putting in place a home death care plan was associated with reductions in hospitalisation or emergency department visits in the last 6 months of life; both interventions increased the likelihood of patients dying in the community. However, this study did not account for confounding variables, such family support and preferences regarding hospital admissions. It is possible that anticipatory prescribing serves as a proxy indicator of the healthcare team’s awareness of the imminence of death and their planning for community-based care.

## Discussion

### Summary of findings

This systematic literature review identified the following key findings about anticipatory prescribing in the community:

1. Prescribing practices vary in relation to community setting, proximity of prescriptions to death and patient populations. The standardised prescribing of four medications for anticipated symptoms is commonplace in the UK; evidence of practices in other countries remains limited. There is limited reliable data on how often medications are administered in the community.
2. Only one small study has directly investigated the experiences or views of patients. The prescribing of anticipatory medications appears to be a significant event for patients and signifies the imminence of death. Further research is urgently needed.
3. Anticipatory prescriptions are accepted by family caregivers despite inadequate explanations and they generally appreciate having access to medications; family caregivers also express ambivalence regarding the helpfulness of medication and have safety concerns. Family caregivers who take on the role of administering anticipatory medications appreciate being able to provide symptom relief, although some struggle with assessing symptoms and worry injections may hasten death.
4. Healthcare professionals perceive that anticipatory prescriptions enable effective symptom control, helps prevent crisis hospital admissions and provide reassurance for everyone involved. Effective teamwork plays a central role in the prescription and use of anticipatory medication. GPs and nurses also express safety concerns and nurses struggle with decisions to start injections and when adjusting doses.
5. Robust evidence of clinical effectiveness remains absent. Two studies suggest the intervention may contribute to symptom relief.
6. Anticipatory prescribing is a relatively low-cost intervention, although most medications appear to go unused. Robust evidence of cost-effectiveness remains absent.

This review identifies the increase in high-quality studies concerning anticipatory prescribing since our original review in 2017 (table 1). However, most studies in both reviews reported on single sites or sampled from populations receiving specialist palliative care input, limiting the generalisability/transferability of findings. In this synthesis, we found similar numbers of papers to the original review, although over a much shorter publication period. This indicates there has recently been increased research interest in anticipatory prescribing. Evidence was limited to the UK, Norway, Australia, New Zealand and Canada. Although anticipatory prescribing is considered best practice internationally,^2 3 11 16 57^ published empirical research from other countries remains rare.

**Table 1:** Number of papers included in the current synthesis and original review

Review question	Number of papers in the current review answering each research question and overall WoE (D) scores	Number of papers in original review^23^ answering each research question and overall WoE (D) scores*
What is current practice?	21 papers: 6 high, 11 medium and 4 low WoE (D) scores	26 papers: 3 high, 16 medium and 7 low WoE (D) scores
What are the attitudes of patients?	1 paper: high WoE (D) score	No papers on patient views or experiences. 2 papers refer to practitioner interpretations of patient views: 1 medium and 1 low overall WOE (D) scores
What are the attitudes of family caregivers?	9 papers: 2 high, 5 medium and 2 low WoE (D) scores	5 papers: 2 medium and 3 low overall WoE (D) scores
What are the attitudes of community healthcare professionals?	15 papers: 5 high, 8 medium and 2 low WoE (D) scores	21 papers: 3 high, 13 medium and 5 low overall WoE (D) scores
What is its impact on patient comfort and symptom control?	2 papers: 1 high and 1 medium WoE (D) scores	3 papers: 2 medium and 1 low overall WoE (D) scores
Is it cost-effective?	3 papers: 3 medium WoE (D) scores	9 papers: 6 medium and 3 low overall WoE (D) scores

*Studies from the original review^23^ were not included in the results and narrative synthesis: they are detailed in the table to give an overview of the overall evidence base.

WoE, Weight of Evidence.

Grey literature was not included in this review. Consequently, our methods did not capture non-peer-reviewed evidence; a methodological decision was made to include only peer-reviewed publications, replicating the original review inclusion and exclusion criteria.^23^ Conference abstracts were included and offer useful insights into care, although all scored WoE low to medium due to limited information on their methods. Conference abstracts accounted for the majority of studies found through journal hand searches: these were not recorded in the nine databases. This highlights the value of the complementary search strategies.

It was difficult to separate anticipatory prescribing before symptoms arise from reactive prescribing after symptoms occur in several studies.^8 17 41^ Two reviewers independently applied the definition of ‘the prescribing of injectable medications ahead of need to control terminal symptoms’^1 3^ and reached consensus by discussion when eligibility was unclear. Studies were excluded when their methods reported on the prescribing and use of injectable end-of-life medications more generally.^58–61^

The evidence underpinning anticipatory prescribing practice and policy remains based primarily on healthcare professionals’ perceptions and experiences that the intervention is reassuring and provides effective, timely symptom relief. However, healthcare professionals also introduce the subject of anticipatory prescribing in a way that plays down the purpose and significance of prescriptions, expecting patients and families to be cautious about having injectable medications, including strong opioids, in the home.^6 25 45 47^ The limited research to date exploring the views and experiences of patients and family caregivers suggest that the intervention is both reassuring and a source of concern.^6 47 50–52^ Only one published study to date, identified in this review, has explored patients’ views regarding anticipatory prescriptions.^47^ Practice and policy based primarily on healthcare professions’ perceptions risks misunderstanding how anticipatory prescriptions are viewed by other key stakeholders.

Patient and family caregiver experiences of anticipatory prescriptions and their involvement in decisions to administer medication with nurses require urgent investigation.^20 25 47 62^ This was also a key recommendation from the original systematic review findings; this priority area has still received inadequate research attention, possibly due to ethical concerns about interviewing dying patients and their families. Recent published research has focused primarily on the views of bereaved family caregivers of patients who received *specialist* palliative care^50–52^; many patients and families do not receive this level of specialist input and care, and may have diverse experiences. We have recently completed in-depth longitudinal interviews exploring patients’, family caregivers’ and their healthcare professionals’ views and experiences of decisions to prescribe and use of anticipatory medications.^63^

Having access to anticipatory medications may not, by itself, adequately resolve the issues the intervention sets out to address: ensuring timely, effective symptom control in the community.^1 64^ Building on the knowledge from our earlier review, it is now clear anticipatory prescribing is a complex intervention involving multiple steps, several layers of teamwork and nuanced, skilled judgements about both when to prescribe and use medication.^7 15 20 25 36 39 45^ These processes are prone to miscommunication and adverse patient safety events, especially when multiple healthcare professionals and services are involved.^15 61 65 66^ Studies have repeatedly identified that families are unsure when to access professional help with symptom control^62 67 68^; when they do, they report experiencing difficulties in getting nurses to visit to administer injectable medications in a timely way.^9 50 52^ Community nurses also struggle with decisions to administer anticipatory medications and less experienced nurses’ report being over-cautious when giving injections, fearing causing over-sedation or hastening death.^7 39^ Paramedic experiences of using anticipatory medications remain unknown and require exploration as the workforce is involved in crisis end-of-life symptom management at home.^69–71^ How lone-working nurses and paramedics can be best supported in assessing risk and making skilled, nuanced decisions to use anticipatory medications warrant careful consideration in practice and policy.

Prior to the start of the COVID-19 pandemic, it was very unusual for family caregivers in the UK to give injectable anticipatory medications, whereas it is routine practice in parts of Australia and New Zealand.^8 23 28^ Different approaches reflect accepted healthcare norms and practices rather than being attributable to the rurality and remoteness of settings. Healthcare responses to the pandemic have accelerated the possibility that family caregivers might take on the role of giving anticipatory medications with suitable training and clinical support.^10 19 20 72^ Findings from the current review highlight this must be considered carefully in practice on a case-by-case basis rather than being used to manage deficits in over-stretched community healthcare services.^9 46^ Family and friends should not perceive that they are obligated or under any pressure to take on this additional responsibility at a particularly stressful time^9 28 67 73^; the alternative of nurse administered injectable medications should be discussed alongside this possibility. Families are vulnerable to the pressure of professional persuasion to take on additional responsibilities in managing symptoms, even if inadvertent. The potential benefits and emotional burdens for family caregivers taking on the role of administering anticipatory medications also needs further investigation and detailed evaluations or what works, when and why before it becomes widespread international practice and policy.

Our current review findings emphasise the need for prospective clinical trials that investigate the impact of anticipatory prescribing on patient symptom control and rates of crisis hospital admissions. Despite consensus guidance promoting individualised prescribing based on likely needs and patient preferences,^1 16^ our current review found evidence that standardised prescriptions of four medications and set dose ranges are now more commonplace and actively promoted by local healthcare systems, at least in some parts of the UK.^5 6 15 42 44 73^ Standardised prescribing recommendations have been shaped by experiences of symptom profiles of patients dying with terminal cancer and may not apply to other health conditions such as dementia. However, standardised prescriptions may have their place. Prescribing medication for future unknowns, often weeks in advance,^6 26 45 47^ is an inherently uncertain process, especially if patients have not previously experienced symptoms that have required strong opioids or sedatives.^15 24^ Prescribing standardised, small starting doses and ranges enables the trialling of anticipatory medications, with subsequent prescriptions adjusted to individual need; whether standardised prescriptions of four medications and set doses are appropriate, regularly reviewed and tailored to individual need once medications are commenced remains unknown. Research and nuanced clinical guidance to improve tailored anticipatory prescribing clinical decision-making is warranted.

Current anticipatory prescribing recommendations are based on inadequate knowledge of the diversity of dying symptom profiles, the clinical effectiveness of prescriptions and their potential adverse effects.^1 15 74^ There is a call in English end-of-life care guidance for a cluster-randomised control trial^1^ that compares the clinical effectiveness of anticipatory prescriptions against prescription in response to symptoms. This may pose a challenge, as anticipatory prescribing is widespread established practice in several countries. However, different types of anticipatory prescribing practices could be compared, including standardised prescribing versus individualised prescribing.

## Conclusion

This systematic review highlights that anticipatory prescribing remains recommended and widespread community practice in the UK, and several other countries, despite a limited evidence base. The evidence underpinning anticipatory prescribing practice and policy remains based primarily on healthcare professionals’ perceptions and experiences that the intervention offers reassurance and provides effective, timely symptom relief in the community. There is still limited evidence concerning likely symptom profiles and which medications and dose ranges are needed. The views and experiences of patients and their family caregivers towards anticipatory prescribing need further investigation. Urgent research is necessary to investigate the clinical effectiveness, cost-effectiveness, safety and acceptability of different anticipatory prescribing practices.

## Data Availability

All data relevant to the study are included in the article or uploaded as supplementary information. No previously unpublished primary data are included in the paper. All data relevant to the systematic review are included in the paper or uploaded as supplementary information.
