# Supplementary material for: Anticipatory prescribing in community end-of-life care: systematic review and narrative synthesis of the evidence since 2017
Source: BMJ Support Palliat Care. 2023 May 26;13(e3):e612–23. doi: 10.1136/spcare-2022-004080 (PMC10850730; doi:10.1136/spcare-2022-004080)
Supplement: Supplementary data [file spcare-2022-004080supp001.pdf]

Supplemental Material 1

Boolean search strategy for the nine databases

All searches were run 1st March 2022

Medline via Ovid

Epub Ahead of Print, In-Process & Other Non-Indexed Citations, Ovid MEDLINE(R) Daily and Ovid MEDLINE(R) May 2017 to March 2022

((palliative adj medicine adj kit*) or (liverpool adj care adj pathway*) or ((end adj2 life) adj2 ((care adj plan*) or (care adj pathway*))) or (gold adj standard* adj framework*) or ((prescrib* or prescription* or medicat* or medicine* or drug* or pharma or pharmaceutical* or packet* or pack* or pak* or box* or kit* or (care adj plan*) or (core adj "4") or (core adj four)) adj3 (crisis* or comfort* or anticipate* or anticipatory or anticipation or preemptive or pre-emptive or (just adj in adj case) or PRN or (pro adj re adj nata) or (as adj required)))).ti,ab. and (exp Terminal Care/ or exp Palliative Care/ or exp "Hospice and Palliative Care Nursing"/ or exp death/ or exp Palliative Medicine/ or exp Terminally Ill/ or ((end adj2 life) or ((final* or last*) adj1 (hour* or day* or minute* or week* or month* or moment*)) or palliat* or terminal* or (end adj stage) or dying or (body adj2 (shutdown or shut* down or deteriorat*)) or deathbed).ti,ab.)

Embase via Ovid

May 2017 to March 2022

((palliative adj medicine adj kit*) or (liverpool adj care adj pathway*) or ((end adj2 life) adj2 ((care adj plan*) or (care adj pathway*))) or (gold adj standard* adj framework*) or ((prescrib* or prescription* or medicat* or medicine* or drug* or pharma or pharmaceutical* or packet* or pack* or pak* or box* or kit* or (care adj plan*) or (core adj "4") or (core adj four)) adj3 (crisis* or comfort* or anticipate* or anticipatory or anticipation or preemptive or pre-emptive or (just adj in adj case) or PRN or (pro adj re adj nata) or (as adj required)))).ti,ab. and (exp *Terminal Care/ or exp *Palliative therapy/ or exp *palliative nursing/ or exp *palliative treatment/ or exp *hospice care/ or exp

Supplemental Material 1

Boolean search strategy for the nine databases

*hospice/ or exp *Terminally Ill Patient/ or exp *dying/ or ((end adj2 life) or ((final* or last*) adj1 (hour* or day* or minute* or week* or month* or moment*)) or palliat* or terminal* or (end adj stage) or dying or (body adj2 (shutdown or shut* down or deteriorat*)) or deathbed).ti,ab.)

CINAHL via Ebsco

TI ((“palliative medicine kit*”) or (“liverpool care pathway*”) or ((end N2 life) N2 ((“care plan*”) or (“care pathway*”))) or (“gold standard* framework*”) or ((prescrib* or prescription* or medicat* or medicine* or drug* or pharma or pharmaceutical* or packet* or pack* or pak* or box* or kit* or (“care plan*”) or (“core 4”) or (“core four”)) N3 (crisis* or comfort* or anticipate* or anticipatory or anticipation or preemptive or pre-emptive or (“just in case”) or PRN or (“pro re nata”) or (“as required”)))) or AB ((“palliative medicine kit*”) or (“liverpool care pathway*”) or ((end N2 life) N2 ((“care plan*”) or (“care pathway*”))) or (“gold standard* framework*”) or ((prescrib* or prescription* or medicat* or medicine* or drug* or pharma or pharmaceutical* or packet* or pack* or pak* or box* or kit* or (“care plan*”) or (“core 4”) or (“core four”)) N3 (crisis* or comfort* or anticipate* or anticipatory or anticipation or preemptive or pre-emptive or (“just in case”) or PRN or (“pro re nata”) or (“as required”))))

And

TI ((end N2 life) or ((final* or last*) N1 (hour* or day* or minute* or week* or month* or moment*)) or palliat* or terminal* or (“end stage”) or dying or (body N2 (shutdown or shut* down or deteriorat*)) or deathbed) or AB ((end N2 life) or ((final* or last*) N1 (hour* or day* or minute* or week* or month* or moment*)) or palliat* or terminal* or (“end stage”) or dying or (body N2 (shutdown or shut* down or deteriorat*)) or deathbed) or (MH "Terminal Care+") OR (MH "Palliative Care") OR (MH "Hospice and Palliative Nursing") OR (MH "Hospice Patients") OR (MH "Hospices") OR (MH "Hospice Care") OR (MH "Terminally Ill Patients") OR (MH "Death+")

PsycINFO via Ebsco

TI ((“palliative medicine kit*”) or (“liverpool care pathway*”) or ((end N2 life) N2 ((“care plan*”) or (“care pathway*”))) or (“gold standard* framework*”) or ((prescrib* or prescription* or medicat* or

Supplemental Material 1

Boolean search strategy for the nine databases

medicine* or drug* or pharma or pharmaceutical* or packet* or pack* or pak* or box* or kit* or (“care plan*”) or (“core 4”) or (“core four”)) N3 (crisis* or comfort* or anticipate* or anticipatory or anticipation or preemptive or pre-emptive or (“just in case”) or PRN or (“pro re nata”) or (“as required”)))) or AB ((“palliative medicine kit*”) or (“liverpool care pathway*”) or ((end N2 life) N2 ((“care plan*”) or (“care pathway*”))) or (“gold standard* framework*”) or ((prescrib* or prescription* or medicat* or medicine* or drug* or pharma or pharmaceutical* or packet* or pack* or pak* or box* or kit* or (“care plan*”) or (“core 4”) or (“core four”)) N3 (crisis* or comfort* or anticipate* or anticipatory or anticipation or preemptive or pre-emptive or (“just in case”) or PRN or (“pro re nata”) or (“as required”))))

And

TI ((end N2 life) or ((final* or last*) N1 (hour* or day* or minute* or week* or month* or moment*)) or palliat* or terminal* or (“end stage”) or dying or (body N2 (shutdown or shut* down or deteriorat*)) or deathbed) or AB ((end N2 life) or ((final* or last*) N1 (hour* or day* or minute* or week* or month* or moment*)) or palliat* or terminal* or (“end stage”) or dying or (body N2 (shutdown or shut* down or deteriorat*)) or deathbed) or ((DE "Terminally Ill Patients") OR (DE "Palliative Care")) OR (DE "Death and Dying" OR DE "Euthanasia" OR DE "Parental Death") OR (DE "Hospice")

Web of Science Core Collection

((“palliative medicine kit*”) or (“liverpool care pathway*”) or ((end near/2 life) near/2 ((“care plan*”) or (“care pathway*”))) or (“gold standard* framework*”) or ((prescrib* or prescription* or medicat* or medicine* or drug* or pharma or pharmaceutical* or packet* or pack* or pak* or box* or kit* or (“care plan*”) or (“core 4") or (“core four”)) near/3 (crisis* or comfort* or anticipate* or anticipatory or anticipation or preemptive or pre-emptive or (“just in case”) or PRN or (“pro re nata”) or (“as required”)))) and ((end near/2 life) or ((final* or last*) near/1 (hour* or day* or minute* or week* or month* or moment*)) or palliat* or terminal* or (“end stage”) or dying or deathbed or (body near/2 deteriorat*) or (body near/2 "shut* down") or (body near/2 shutdown))

Supplemental Material 1

Boolean search strategy for the nine databases

Cochrane Library

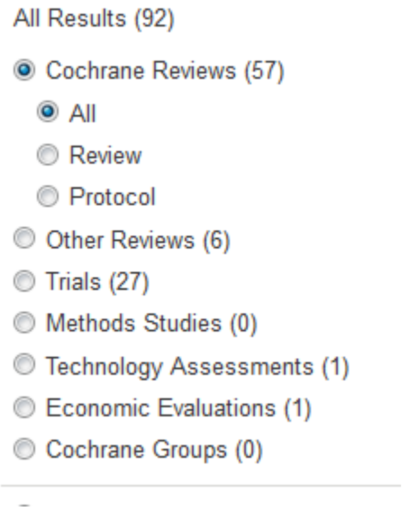

#1 (("palliative medicine kit*") or ("liverpool care pathway*") or ((end near/2 life) near/2 (("care plan*") or ("care pathway*"))) or ("gold standard* framework*") or ((prescrib* or prescription* or medicat* or medicine* or drug* or pharma or pharmaceutical* or packet* or pack* or pak* or box* or kit* or ("care plan*") or ("core 4") or ("core four")) near/3 (crisis* or comfort* or anticipate* or anticipatory or anticipation or preemptive or pre-emptive or ("just in case") or PRN or ("pro re nata") or ("as required"))))

#2 ((end near/2 life) or ((final* or last*) near/1 (hour* or day* or minute* or week* or month* or moment*)) or palliat* or terminal* or ("end stage") or dying or deathbed or (body near/2 deteriorat*) or (body near/2 "shut* down") or (body near/2 shutdown))

#3 MeSH descriptor: [Terminal Care] explode all trees

#4 MeSH descriptor: [Palliative Care] explode all trees

#5 MeSH descriptor: [Hospice and Palliative Care Nursing] explode all trees

#6 MeSH descriptor: [Palliative Medicine] explode all trees

#7 MeSH descriptor: [Death] explode all trees

#8 #2 or #3 or #4 or #5 or #6 or #7

#9 #1 and #8

Supplemental Material 1

Boolean search strategy for the nine databases

Social Care Online

May 2017 to March 2022

"palliative medicine kit" or "liverpool care pathway" or "end of life" and care plan or care pathway or "gold standard framework"

And

"palliative medicine kit" or "liverpool care pathway" or "end of life" and care plan or care pathway or "gold standard framework"

"palliative medicine kit" or "liverpool care pathway" or "end of life care plan" or “end of life care pathway” or "gold standard framework"

And

palliative or terminal or death or dying or last or final or "end stage" or “body shutdown” or “body shut down”

title

medicine or drug or prescription or prescribing or medication or "core 4" or "core four" or packet or pack or box or kit or "care plan"

and

palliative or terminal or death or dying or last or final or "end stage" or body shutdown or body shut down

and

crisis or comfort or comfortable or anticipate or anticipatory or ancticipation or preemptive or pre-emptive or "just in case" or PRN or "pro re nata" or "as required"

Supplemental Material 1

Boolean search strategy for the nine databases

HMIC via Ovid

((palliative adj medicine adj kit*) or (liverpool adj care adj pathway*) or ((end adj2 life) adj2 ((care adj plan*) or (care adj pathway*))) or (gold adj standard* adj framework*) or ((prescrib* or prescription* or medicat* or medicine* or drug* or pharma or pharmaceutical* or packet* or pack* or pak* or box* or kit* or (care adj plan*) or (core adj "4") or (core adj four)) adj3 (crisis* or comfort* or anticipate* or anticipatory or anticipation or preemptive or pre-emptive or (just adj in adj case) or PRN or (pro adj re adj nata) or (as adj required)))).ti,ab. and (exp terminal care/ or exp Terminal nursing/ or exp Terminal illness/ or exp Hospices/ or exp "End of life care"/ or exp Palliative care/ Or exp Death/ or ((end adj2 life) or ((final* or last*) adj1 (hour* or day* or minute* or week* or month* or moment*)) or palliat* or terminal* or (end adj stage) or dying or (body adj2 (shutdown or shut* down or deteriorat*)) or deathbed).ti,ab.)

Kings Fund

Results from May 2017 to March 2022:

The rising cost of medicines to the NHS What's the story?

https://www.kingsfund.org.uk/sites/default/files/2018-04/Rising-cost-of-medicines.pdf

Innovative approaches to end of life care

https://www.kingsfund.org.uk/events/innovative-approaches-end-life-care
