# Supplementary material for: Anticipatory prescribing in community end-of-life care: systematic review and narrative synthesis of the evidence since 2017
Source: BMJ Support Palliat Care. 2023 May 26;13(e3):e612–23. doi: 10.1136/spcare-2022-004080 (PMC10850730; doi:10.1136/spcare-2022-004080)
Supplement: Supplementary data [file spcare-2022-004080supp002.pdf]

Supplemental Material 2

Data Extraction Tool

**Details of publication**	
• First author	
• Reference	
**Introduction**	
• Aims	
**Study participants**	
• Country of study	
• Recruitment	
• Characteristics of participants:<br>- Number<br>- Setting (home, hospital etc)<br>- Age / sex / social class / ethnicity	
**Methods**	
• Date of fieldwork	
• Research methods	
• Analysis	
**Key findings relevant to review**	
1) What is **current practice?**<br>*Who prescribes, for whom, proximity to death?*<br>*Who administers?*<br>*Absence of AP?*	
2) What are the **attitudes of patients** to AP?<br>*Patients' acceptance / views*	
3) What are the **attitudes of family caregivers** to AP?<br>*Carers' acceptance / views*	
4) What are the **attitudes of community healthcare professionals** to AP?<br>*HCPs' acceptance of AP, concerns, views re use, barriers, facilitators, etc.*	

Supplemental Material 2

Data Extraction Tool

5) Evidence for **clinical effectiveness**?<br><br>***Clinical:*** *comfort / symptom control (who reported)*	
6) Evidence for **cost / cost-effectiveness**?<br><br>***Service use:*** *admission avoidance, place of death, healthcare activity, etc.*<br><br>***Cost****: costs of drugs, admissions, healthcare activity, ect*	
**Author(s) conclusion(s)**	
**Reviewer assessment of the internal validity, appropriateness and contribution of the study in answering the relevant research questions (Gough's WoE)**	
**Weight of Evidence A**<br><br>Coherence and integrity of the evidence *in its own terms*	
**Weight of Evidence B**<br><br>Appropriateness *of form of evidence* for answering review question	
**Weight of Evidence C**<br><br>*Relevance of the evidence* for answering review question	
**Weight of Evidence D**<br><br>*Overall assessment* of study contribution to answering review question	
