# Supplementary material for: Anticipatory prescribing in community end-of-life care: systematic review and narrative synthesis of the evidence since 2017
Source: BMJ Support Palliat Care. 2023 May 26;13(e3):e612–23. doi: 10.1136/spcare-2022-004080 (PMC10850730; doi:10.1136/spcare-2022-004080)
Supplement: Supplementary data [file spcare-2022-004080supp003.pdf]

Supplemental Material 3

Summary of included studies

Author and country	Participants	Study aims and methods	Key findings	Weight of evidence<br><br>A +B +C = D
Staats et al. (2018) [1]<br><br>Norway	18 community and cancer care nurses working in one region	To increase the understanding concerning community nurse experience with anticipatory medication in symptom management for the terminally ill<br><br>Methods: Qualitative interviews and focus groups. Qualitative analysis	• The assessment of symptoms and when to administer prescribed drugs is delegated to nurses<br>• Good communication and meetings with GPs deemed essential in facilitating the appropriate use of medication<br>• Recently qualified nurses did not feel confident in assessing the need for medication<br>• Nurses worked alone mainly, this caused great variation in medication kit administration<br>• There was vulnerability felt in relation to using medication kits at night<br>• Nurses felt more confident continuing a dose that had been started by the day staff than being the one to initiate the medication for the first time	H H H – H
Bowers et al. (2022) [2]<br><br>UK	329 deceased patients with 12 GP practices in two counties	To investigate the frequency, timing and recorded circumstances of anticipatory medication prescribing for patients living at home and in residential care<br><br>Methods: Retrospective notes review. Statistical and qualitative analysis	• 51% prescribed anticipatory medication, between 0 and 1212 days (median 17 days) before death<br>• The likelihood of AMs prescribing was significantly higher for patients with a recorded preferred place of death (OR 34; 95% CI 15–77; $p < 0.001$) and specialist palliative care involvement (OR 7; 95% CI 3–19; $p < 0.001$)<br>• Most patients (92%) were prescribed anticipatory medications for all five common end-of-life symptoms: pain, breathlessness, nausea and vomiting, agitation and respiratory tract secretions.<br>• Standardised prescribing was commonplace and prompted by primary care electronic end-of-life templates (63% of the patients prescribed medications)	H H H – H

Supplemental Material 3

Summary of included studies

Author and country	Participants	Study aims and methods	• Key findings	Weight of evidence<br><br>A +B +C = D
Bowers et al. (2020) [3]<br><br>UK	13 GPs working in two counties	To explore GPs' decision-making processes in the prescribing and use of anticipatory medications for patients at the end of life<br><br>Methods: Qualitative interviews. Qualitative analysis	• GPs generally prescribed drugs while patients were relatively stable, as it helped them manage the uncertainty<br>• The prescribing of anticipatory medications was recognised as a harbinger of death for patients and their families<br>• GPs often presented anticipatory medications as a clinical recommendation to ensure patients and families accept the prescription<br>• In some cases, prescribed drugs remained in the home for months or went unused<br>• GPs relied on nurses to assess when to administer drugs and keep them updated about their use: easy access to one another and good communication was perceived to be crucial	H H H – H
Poolman et al. (2020) [4]<br><br>UK	40 patient, family caregiver dyads from three regions; 22 completed the follow-up visit.<br><br>Interviews: 12 bereaved family caregivers; 20 healthcare professionals: 3 GPs; 14 community nurses; and 3 specialist palliative care nurses	To assess if family caregiver administration of as-needed injectable medication for common breakthrough symptoms in patients dying at home is feasible and acceptable<br><br>Methods: Multicentre randomised control pilot trial, including qualitative interviews with family caregivers and healthcare professionals. Descriptive statistics and qualitative analysis	• Family care confidence in administering medication increased over time; family caregivers required different amounts of training to feel confident<br>• The intervention was acceptable to family caregivers, who found it helpful and reassuring<br>• The median time to administer medication in the intervention group was 5 minutes verses 105 minutes for the usual-care group<br>• Many caregivers in the study intervention arm had previous healthcare training<br>• Caregivers worried about accidentally hastening death<br>• Clinicians had a positive view of the intervention in terms of its effects on symptom management and benefits<br>• Clinicians were very careful about who to approach to take part and were concerned about potential family caregiver distress re giving the 'last injection' before death	H H M – H

Supplemental Material 3

Summary of included studies

Author and country	Participants	Study aims and methods	• Key findings	Weight of evidence<br><br>A +B +C = D
Pollock et al. (2021) [5]<br><br>UK	Workstream one: 21 bereaved family caregivers (13 had experience of anticipatory medications). 40 healthcare professionals: 16 palliative care nurses; 8 community nurses; 3 specialist nurses; 7 GPs; 4 pharmacists; and 2 consultants.<br><br>Workstream two: 21 patient cases, each of which included 1-5 participants (6 case study participants had experience of anticipatory medications): 15 patients; 19 family caregivers; 14 healthcare professionals.	To explore how patients, their family caregivers and the healthcare professionals who support them engage in the tasks of managing complex medication regimens and routines of care for patients who are approaching the end of life at home<br><br>Methods: Qualitative interviews and focus groups, interviews over time based on patient cases, observations and medical records review (8 cases). Qualitative analysis	• 46% of family caregivers in workstream one reported anticipatory medications were used<br>• Clinicians preferred to prescribe medication well in advance of anticipated need or even when there was not a strong likelihood that they would be needed<br>• Anticipatory prescribing was a significant event for patients and their families, clearly signifying the imminence of death<br>• Family caregivers valued the availability of anticipatory medication when they were needed<br>• Several family caregivers reported medication were prescribed without accompanying explanation or discussion<br>• Some participants stored the drugs out of sight to keep them safe so that they were not reminded of their purpose<br>• Clinicians tended to be vague and avoided opportunities for providing explicit information<br>• Family caregivers worried about the storage of controlled drugs in the house and their role in administering these to patients	H H M – H

Supplemental Material 3

Summary of included studies

**Author and country**	**Participants**	**Study aims and methods**	• **Key findings**	**Weight of evidence**<br><br>**A +B +C = D**
Antunes et al. (2020) [6]<br><br>UK and Ireland<br><br>*(Completed at the start of the Covid-19 pandemic: April 2020)*	261 palliative care doctors, GPs, community nurses, clinical nurse specialists, pharmacists and other professional groups	To investigate clinicians' experiences concerning changes in anticipatory prescribing during the Covid-19 pandemic and their recommendations for change<br><br>Methods: Survey with open and closed questions. Descriptive and qualitative analysis	• Reported changes in practice related to possible administration by family or social caregivers and drug availability<br>• At the same time, clinical contact and patient assessment were changing to telephone or video rather than in person<br>• Fear of waste and cost are factors that limit the amount of anticipatory prescribing in the community<br>• Having access 24 hours for anticipatory medication prescriptions and drugs in key in enabling rapid response and symptom control	M H H – H
Morgan, et al., (2022) [7]<br><br>UK	164 deceased patients prescribed anticipatory medications, registered with 12 GP practices in two counties	To identify the prescription, usage and wastage costs of anticipatory medications for patients living at home and in residential care<br><br>Methods: Retrospective notes review. Statistical analysis	• Median anticipatory prescription cost was £43.17 (IQR: £38.98-£60.47, range £8.76 to £229.82)<br>• Median administration prescription cost was £2.16 (IQR: £0.00-£12.09, range £0.00 to £83.14)<br>• Median wastage was £41.47 (IQR: £29.15-£54.33, range £0.00 to £195.36)<br>• Haloperidol and cyclizine, contributed 49% of total wastage costs	M H M – M
Ryan et al. (2020a) [8]<br><br>UK	89 healthcare professionals from across the UK: 25 palliative care nurses; 24 palliative consultants; 22 GPs, community nurses and pharmacists	To explore the views of UK healthcare professionals about best practice and areas in need of improvement in anticipatory prescribing<br><br>Methods: Focus groups and survey. Descriptive statistics and qualitative analysis	• 38% were confident that anticipatory prescribing was done well<br>• 20% were concerned about unsafe practice<br>• Top-tips for achieving practice included reducing cross-system complexity by unifying documents and electronic systems	M H M – M

Supplemental Material 3

Summary of included studies

Author and country	Participants	Study aims and methods	Key findings	Weight of evidence<br><br>A +B +C = D
Pilsworth et al. (2021) [9]<br><br>UK	18 bereaved family caregivers receiving care from one specialist palliative care team	To explore family caregivers experiences of anticipatory prescribing and identify ways to improve practice<br><br>Methods: Qualitative interviews. Qualitative analysis	• Some family caregivers reported feeling shocked and distressed when they realised that the medications indicated that their relative was approaching end of life<br>• Concerns associated with obtaining, storing and eventually disposing of medications<br>• Systems barriers, including sourcing the right professional support in a timely manner to administer medication often proved problematic	M H M – M
Johnston et al. (2019) [10]<br><br>Australia	40 staff in one area: 20 carers; 13 nurses; 4 team leaders; 2 managers; 1 geriatrician<br><br>*(findings relate to these participants)*	To understand the experience and impact of integrating a specialist palliative care model on care homes residents, relatives and staff<br><br>Methods: Qualitative interviews. Qualitative framework analysis	• Perception of care home staff that anticipatory prescribing done more because of having palliative care nurse practitioner input in identifying and reviewing deteriorating patients<br>• Prescriptions perceived as a useful tool for preventing hospital admissions<br>• GP-nurse trust crucial in prescribing and use: trust between GPs and the registered nurses at the facilities improved with specialist palliative nurse input	M H M – M

Supplemental Material 3

Summary of included studies

Author and country	Participants	Study aims and methods	Key findings	Weight of evidence<br><br>A +B +C = D
Healy et al. (2018) [11]<br><br>Australia	93 Family caregivers, allocated to one of three intervention arms in a large region: group 1: 27; group 2: 30; group 3: 36	To explore differences in laycarers' confidence in administering subcutaneous injections depending on whether an family caregiver, nurse or pharmacist prepared injections<br><br>Methods: Quasi-randomised control trial. Statistical analysis	• Family caregivers self-reported confidence with experience of administering injections went from 5.3 for the first injection to 6.1 for subsequent injections on a 7-point (7 = extremely confident) Likert scale<br>• Neither the mean level of confidence nor change in confidence over time differed significantly across groups	H M M – M
Cornish and French (2018) [12]<br><br>UK	49 deceased patients on community nursing caseloads in two counties. 20 GPs	To evaluate whether a new community anticipatory medication chart and guidance facilitates safe, appropriate and consistent prescribing<br><br>Methods: Audit of medical records; survey of GPs. Descriptive statistics	• 47 of the 49 expected deaths had an anticipatory medication chart in place<br>• Deceased patients were prescribed: opioid (84%), antiemetic (97%), antisecretory (94%) and anxiolytic (94%)<br>• All GPs surveyed agreed that the new chart facilitates safe and appropriate anticipatory prescribing	M M M – M

Supplemental Material 3

Summary of included studies

Author and country	Participants	Study aims and methods	Key findings	Weight of evidence<br><br>A +B +C = D
Tran et al. (2021) [13]<br><br>UK	76 patients receiving the care of who received care from one specialist palliative care organisation (hospice)	To evaluate anticipatory prescribing practices against current local guidelines<br><br>Methods: Retrospective records review. Descriptive statistics	• All patients were prescribed and dispensed four medications for: pain, agitation, secretions and nausea/vomiting<br>• There was close adherence to local guidelines (choice of drug, dose)<br>• Most commonly prescribed drugs were: midazolam 99%; glycopyrronium 97%; haloperidol 88%; morphine 61%<br>• 64% had stats given at end-of-life: 53% for pain, 41% for agitation, 24% for secretions; 16% for nausea<br>• Community nurses and paramedics administered the medications<br>• All four medications cost approximately £50 per patient: haloperidol accounted for 60% of costs and was not often used	M H L – M
Rainbow and Faull (2017) [14]<br><br>UK	50 deceased patients registered with one GP practice	To describe the prescribing and usage of anticipatory medications in the community<br><br>Methods: Retrospective notes review. Descriptive statistics	• 44% of deceased patients prescribed anticipatory medication<br>• Medication issued by diagnosis: cancer 10/16 (62%), frailty/dementia 11/22 (50%), sudden death 1/5 (20%)<br>• Median number of days AM issued and started [X] before death: cancer 14[4], frailty/dementia 6[4], sudden death 11[6]	M M M – M
Hedges et al. (2021) [15]<br><br>UK<br><br>*(Completed during the first year of the Covid-19 pandemic: 2020)*	8 bereaved family caregivers receiving care from one specialist palliative care team	To explore bereaved family caregivers' experiences, feelings and perspectives relating to when a family member was prescribed anticipatory medications at home<br><br>Methods: Qualitative interviews. Qualitative analysis	• Anticipatory medications were accepted in the home, despite inadequate explanation, because symptoms or suffering were expected<br>• Medications did not have the presumed effect: there was a perception of lack of benefit and harm	M H L – M

Supplemental Material 3

Summary of included studies

Author and country	Participants	Study aims and methods	Key findings	Weight of evidence<br><br>A +B +C = D
Hughes et al. (2021) [16]<br><br>UK	38 bereaved family caregivers who received care from one specialist palliative care organisation (hospice or community)	To explore family caregivers' experiences of anticipatory medication and explore ways to improve practice<br><br>Methods: Survey 3-9 months after death. Descriptive statistics and qualitative analysis	• 87% of respondents said there were benefits of having anticipatory medications available and were reassured by their presence<br>• Some people found medicines distressing as they highlighted that death was imminent<br>• 'Just over half' of the respondents reported that the medication was used, usually for pain or agitation with good effect<br>• Of the patients who required medications, the problems caregivers reported were deciding when to call for help (21%); delays in clinicians attending to administer medication (29%); knowledge of the clinician attending (24%)	M M M – M
Katz et al. (2019) [17]<br><br>Australia and New Zealand	121 doctors: 104 consultant and 17 trainee palliative care doctors working in two countries	To explore palliative medicine doctors' approaches to pre-emptive prescription of medications to manage catastrophic events<br><br>Methods: Staff Survey. Descriptive statistics	• Clinicians prescribe crisis medication to prevent poor symptom control and unrelieved distress<br>• The most commonly prescribed crisis management drugs were morphine midazolam<br>• 25% of clinicians reported being aware of adverse outcomes due to medications being prescribed for potential catastrophic events<br>• 50% were aware of adverse events related to medications not being prescribed<br>• Many clinicians reflected on a lack of evidence, a desire for further studies and standardised approaches to support practice<br>• Concerns about prescribing: discussing an unlikely event can cause disproportionate and often unnecessary anxiety; it can be challenging to know when to administer medications	M M M – M

Supplemental Material 3

Summary of included studies

Author and country	Participants	Study aims and methods	Key findings	Weight of evidence<br><br>A +B +C = D
Ponnampa-lampillai et al. (2018) [18]<br><br>UK	132 deceased patients who accessed a county-wide community palliative care co-ordination centre	To evaluate anticipatory prescribing across one county<br><br>Methods: Retrospective notes review. Descriptive statistics	• Recognition of the need for prescribing came from palliative care nurses (50%), GPs (32%) and community nurses (14%)<br>• Median timing between anticipatory prescription and first drug administration was 9 days for patients with cancer (range 0 to 368 days), and 61 days for those with non-cancer conditions (range 3 to 298 days)<br>• 37% of medications prescribed were administered	M M M – M
Khalil et al. (2018) [19]<br><br>Australia	29 community nurses and palliative care nurses	To identify the challenges with the administration and access to anticipatory medications in rural and remote community settings<br><br>Methods: Staff Survey. Descriptive statistics	• Opioids (55%) were reported as the most commonly used anticipatory medication followed by antiemetics (45%), clonazepam (41%) and midazolam (41%)<br>• Most thought it was useful to organise medications in the home<br>• Barriers to prescribing: a third of all nurses indicated that doctors were not willing to prescribe drugs on some occasions due to the fear of drug misuse and/or abuse<br>• Reported issues in using drugs: lack of confidence about usage and doses; pharmacy shortages; inability to access medications<br>• Reported incidents included giving the wrong dose of medication and expired medications given	M M M – M
Rainbow (2017) [20]<br><br>UK	16 participants: 5 community palliative care nurses; 4 hospice at home nurses; 1 community nurse; 3 GPs; 1 community pharmacist; 2 relatives	To investigate experiences of prescribing, administering, dispensing and observing anticipatory medication at the end of life<br><br>Methods: Qualitative interviews. Qualitative analysis	• Anticipatory prescribing and standardised systems were felt to have improved the management symptoms at the end of life	M H L – M

Supplemental Material 3

Summary of included studies

Author and country	Participants	Study aims and methods	Key findings	Weight of evidence<br><br>A +B +C = D
Benson et al. (2021) [21]<br><br>UK	347 deceased patients under the care of 174 hospital, 49 community and 124 hospice teams (1 patient per site)	To identify the use of syringe pumps (drivers) across a network<br><br>Methods: Retrospective notes review and network discussion of results. Descriptive statistics and description	• 58% of services responding to the survey allowed anticipatory syringe pump prescribing<br>• 33% of patients in the community prescribed anticipatory syringe pumps<br>• Two conflicting sets of views and practices regarding anticipatory syringe pumps: some clinicians considered them vital to ensure timely symptom; others viewed it at unsafe practice, citing incidents / near-misses resulting from lack of clinical assessment of need when syringe pump started	M M L – M
Coyle et al. (2021) [22]<br><br>UK	223 deceased patients prescribed anticipatory syringe in part of one county during a 12-month period	Audit of anticipatory syringe driver prescription and administration practices, benchmarked against local guidance<br><br>Methods: retrospective notes review. Statistical analysis and benchmarking care against local guidance	• 136/223 (61%) of anticipatory syringe pumps prescribed were used<br>• None the 97/213 cases where midazolam was administered were considered unsafe<br>• Only the dosages used for one of the 115 patients who received opioid administration was considered unsafe<br>• Cyclizine administered to 40/158 people; antisecretories administered to 57/206 people<br>• Midazolam was the only benzodiazepine given: ranges of prescription were more likely to outside of the range stated within guidance if had SPC input ($p=0.04$) and more likely to have it administered ($p<0.0001$)	M M M – M

Supplemental Material 3

Summary of included studies

Author and country	Participants	Study aims and methods	Key findings	Weight of evidence<br><br>A +B +C = D
Webber et al. (2019) [23]<br><br>Canada	Population level study = 5223 patient deaths<br><br>Retrospective cohort study = 4538 patient deaths	To evaluating the impact of a home medication kit and home-death planning tool on place of death, hospitalisations, and emergency department visits among palliative home care patients<br><br>Methods: Population-level and retrospective cohort study using medical records. Statistical analysis	• Compared with patients who received neither intervention, patients who received the home-death planning tool or home medication kit had an increased likelihood of dying in the community, with the largest relative risk observed in patients who received both interventions<br>• Receipt of these interventions was only associated with reductions in hospitalisation or emergency department visit rates in the six months of life	M M L – M
Ryan et al. (2020b) [24]<br><br>UK	Anticipatory prescribing guidance documents from 49 areas of the UK: 5 national (representing all 4 countries) and 44 local (33 English, 11 Scottish)	To investigate the scope and content of UK anticipatory prescribing governance documents<br><br>Methods: Qualitative and quantitative content analysis using a previously developed anticipatory prescribing process framework	• Anticipatory prescribing is widespread established practice in the UK, with two typologies of guidance<br>• Type 1: AP guidance is embedded within 'last days of life' symptom management guidelines and is usually limited to the prescribing and administration phases<br>• Type 2: AP guidance covers more than the 'last days of life' period and documents specifically address all 5 phases of the AP process	M L M – L

Supplemental Material 3

Summary of included studies

Author and country	Participants	Study aims and methods	Key findings	Weight of evidence<br><br>A +B +C = D
Khalil et al. (2021) [25]<br><br>Australia	Stage one: 799 patient records for 25 GP practices<br><br>Stage two: 5 palliative care nurse practitioners and GPs	To map the use of end-of-life and anticipatory medications in a cohort of palliative care patients GP medical records and to discuss the results through stakeholder consultation<br><br>Methods: Retrospective note review. Stakeholder interviews. Statistical analysis and qualitative analysis	• 13.5% of patients with a palliative care referral flagged in their records were prescribed injectable or oral end-of-life medications<br>• A referral to specialist palliative care trigger a standard request to GPs for anticipatory medications<br>• Barriers to prescribing: identifying the right stage to prescribe drugs and fears of expediating death<br>• Facilitators for prescribing: good working relationships between nurses and GPs; forward planning approach	L M L – L
Lewis et al. (2021) [26]<br><br>UK	6 family caregivers in one county	Evaluation of a scheme to train family caregivers to give anticipatory medication and the intervention's acceptability to carers<br><br>Methods: Review of patient records and family caregiver feedback. Description	• System in place to train some family caregivers to administer medication (criteria not given)<br>• Drugs started and given between 6 to 137 days before death (median: 9 days)<br>• Data available for four of the six family caregivers trained: all four responded to say training was 'acceptable'	L M L – L
Ward (2020) [27]<br><br>UK	12 organisations anticipatory prescribing guidance in one region (hospices, hospital and community teams)	Determining a baseline of current practice in guidance<br><br>Methods: Document review. Description	• Guidance had a general agreement on which medications to prescribe<br>• 'Small numbers of centres' advise on anticipatory syringe pumps	L M L – L

Supplemental Material 3

Summary of included studies

**Author and country**	**Participants**	**Study aims and methods**	**Key findings**	**Weight of evidence**<br><br>**A +B +C = D**
Dredge et al. (2017) [28]<br><br>Australia	7 community palliative care nurses working for one organisation. A 'small number' of family caregivers	To measure nursing staff satisfaction with changes in anticipatory prescribing practice and early feedback on an educational programme to train family caregivers to administer injectable anticipatory medication<br><br>Methods: Staff survey and verbal feedback from family caregivers. Descriptive statistics	• Change in practice from all patients prescribed anticipatory medications to individual assessment of need by nurses based on agreed criteria (criteria not given).<br>• GP prescribes medication following a request from the specialist palliative care team<br>• Perceived barriers to prescribing included a lack of access to GPs<br>• Medications are administered by nurses or family caregivers with suitable training<br>• Relatively few caregivers both willing and able to undertake education programme<br>• Reports on positive feedback from caregivers on the training to administer drugs	L L L – L

References

[1] Staats K, Tranvåg O, Grov EK. Home-care nurses' experience with medication kit in palliative care: *J Hosp Palliat Nurs* 2018; 20: E1–E9.

[2] Bowers B, Pollock K, Barclay S. Unwelcome memento mori or best clinical practice? Community end-of-life anticipatory medication prescribing practice: a mixed methods observational study. *Palliat Med* 2022; 36: 95–104.

[3] Bowers B, Barclay SS, Pollock K, et al. GPs' decisions about prescribing end-of-life anticipatory medications: a qualitative study. *Br J Gen Pract* 2020; 70: e731–e739.

[4] Poolman M, Roberts J, Wright S, et al. Carer administration of as-needed subcutaneous medication for breakthrough symptoms in people dying at home: the CARiAD feasibility RCT. *Health Technol Assess* 2020; 24: 1–150.

[5] Pollock K, Wilson E, Caswell G, et al. Family and health-care professionals managing medicines for patients with serious and terminal illness at home: a qualitative study. *Health Serv Deliv Res* 2021; 9: 194.

Supplemental Material 3

Summary of included studies

[6] Antunes B, Bowers B, Winterburn I, et al. Anticipatory prescribing in community end-of-life care in the UK and Ireland during the COVID-19 pandemic: online survey. *BMJ Support Palliat Care* 2020; 10: 343–349.

[7] Morgan L, Barclay S, Bowers B. 44 End of life anticipatory medication: understanding the financial costs of medicines. *BMJ Support Palliat Care* 2022; 12: A18.

[8] Ryan R, Spathis A, Bowers B, et al. 82 Anticipatory prescribing in community end of life care in the UK: a mixed-methods study of healthcare professionals' views concerning best practice and areas in need of improvement. *BMJ Support Palliat Care* 2020; 10: A37.

[9] Pilsworth A, Patterson A, Faull C. Dying at 'Home': Bereaved family caregivers' experiences of just-in- case medications at end of life. *Palliat Med* 2021; 35: 48.

[10] Johnston N, Lovell C, Liu W-M, et al. Normalising and planning for death in residential care: findings from a qualitative focus group study of a specialist palliative care intervention. *BMJ Support Palliat Care* 2019; 9: e12–e12.

[11] Healy S, Israel F, Charles M, et al. Laycarers can confidently prepare and administer subcutaneous injections for palliative care patients at home: A randomized controlled trial. *Palliat Med* 2018; 32: 1208–1215.

[12] Cornish C, French S. 73 Introduction of a community palliative care drug chart to facilitate individualised and appropriate anticipatory prescribing. *BMJ Support Palliat Care* 2018; 8: A1-79.

[13] Tran T, Lee C, Ross J. P-141 Anticipatory prescribing at end-of-life: do we need to change practice? *BMJ Support Palliat Care* 2021; 11: A60.

[14] Rainbow E, Faull C. P-120 Describing anticipatory medicine use in the community at the end of life. *BMJ Support Palliat Care* 2017; 7: A52.

[15] Hedges V, Wee B, Bond C. 81 A qualitative study of family carer experience of anticipatory prescribing of injectable medications for end of life care at home. *BMJ Support Palliat Care* 2021; 11: A37.

[16] Hughes H, McEvoy K, Faull C, et al. 1 What is the experience of caregivers in relation to anticipatory medications? *BMJ Support Palliat Care* 2021; 11: A8.

[17] Katz NT, Sacks BH, Le BH, et al. Pre-emptive prescription of medications for the management of potential, catastrophic events in patients with a terminal illness: A survey of palliative medicine doctors. *Palliat Med* 2019; 33: 178–186.

[18] Ponnampalampillai A, Marvin R, Grove S, et al. 74 Anticipatory prescribing in palliative care: evaluation of current practice. *BMJ Support Palliat Care* 2018; 8: A1-79.

[19] Khalil H, Poon P, Byrne A, et al. Challenges Associated with Anticipatory Medications in Rural and Remote Settings. *J Palliat Med* 2018. Online First: 10.1089/jpm.2018.0354.

Supplemental Material 3

Summary of included studies

[20] Rainbow E. P-121 A thematic analysis of anticipatory medicine use in the community at end of life. *BMJ Support Palliat Care* 2017; 7: A52.

[21] Benson S, Cadwallader C, Cartwright-Terry T, et al. 58 Anticipatory prescribing for continuous sub-cutaneous infusion: friend or foe? *BMJ Support Palliat Care* 2021; 11: A1–A63.

[22] Coyle S, Sinden R, Wignall-Coyle J, et al. Continuous subcutaneous infusion: is community anticipatory prescribing and administration safe? *BMJ Support Palliat Care* 2021; bmjspcare-2021-003259.

[23] Webber C, Viola R, Knott C, et al. Community Palliative Care Initiatives to Reduce End-of-Life Hospital Utilization and In-Hospital Deaths: A Population-Based Observational Study Evaluating Two Home Care Interventions. *J Pain Symptom Manage* 2019; 58: 181-189.e1.

[24] Ryan R, Bowers B, Spathis A, et al. 11 How is anticipatory prescribing in community end of life care governed in the UK? A content anaylsis of national and local guidance documents. *BMJ Support Palliat Care* 2020; 10: A4-5.

[25] Khalil H, Garett M, Byrne A, et al. Mapping end-of-life and anticipatory medications in palliative care patients using a longitudinal general practice database. *Palliat Support Care* 2021; Online First: 1–7.

[26] Lewis M, Heneghan J, Burdon J. 107 A service evaluation of a new policy supporting carers giving subcutaneous anticipatory medication at home at the end of life within southern Derbyshire, population of 607,000. *BMJ Support Palliat Care* 2021; 11: A47.

[27] Ward B. 88 A survey of anticipatory prescribing practices in the West Midlands. *BMJ Support Palliat Care* 2020; 10: A39.

[28] Dredge A, Oates L, Gregory H, et al. Effective change management within an Australian community palliative care service. *Br J Community Nurs* 2017; 22: 536–541.
